# Mechanical Circulatory Support in Paraganglioma Induced Cardiogenic Shock and Intestinal Ischemia: Lessons from a Complex Case and Narrative Review

**DOI:** 10.3390/jcm14165882

**Published:** 2025-08-20

**Authors:** Alessio Giordano, Letizia Canu, Manuela Mastronardi, Luisa Petrone, Clotilde Sparano, Mauro Marzano, Carlo Bergamini, Paolo Prosperi

**Affiliations:** 1 Emergency Surgery Unit, Department of Emergency and Acceptance, Careggi University Hospital, 50134 Florence, Italy; mromarzano@gmail.com (M.M.);; 2 Department of Experimental and Clinical Biomedical Sciences “Mario Serio”, University of Florence, 50134 Florence, Italyclotilde.sparano@unifi.it (C.S.); 3 General Surgery Unit, Department of Medicine, Surgery and Health Sciences, University Hospital of Trieste, 34149 Trieste, Italy; manuela.mastronardi@gmail.com; 4 Endocrinology Unit, Medical-Geriatric Department, Careggi University Hospital, 50134 Florence, Italy

**Keywords:** pheocromocytoma, paraganglioma, ECMO, cardiogenic shock, intestinal ischemia

## Abstract

**Background**: The catecholamine-induced hypertensive crisis is a rare, life-threatening condition caused by excessive catecholamine release, often resulting in cardiogenic shock and multiorgan failure. Management is challenging, especially when hemodynamic instability persists despite standard medical therapy. **Methods**: We conducted a narrative review of published articles between 2013 and 2025. The search was conducted in MEDLINE (PubMed, Scholar and Embase). We also presented a case managed at our reference center. **Results**: Overall, 42 studies including 69 patients were included. ECMO was the most commonly used modality, often serving as a bridge to surgery. The overall hospital mortality rate was 17.4%. Timing of adrenalectomy varied, with no clear consensus on the optimal approach. We report also a case of a 43-year-old woman with neurofibromatosis type 1 who developed acute cardiogenic shock due to an adrenal paraganglioma. She was supported with ECMO and underwent emergency bowel resection for intestinal ischemia, followed by adrenalectomy. Despite aggressive treatment, the patient died from progressive multiorgan failure. **Conclusions**: This case highlights the complexity of managing paraganglioma crisis, the potential role of ECMO as a bridge to surgery, and the importance of individualized, multidisciplinary care. Early recognition and referral to specialized centers are essential, though further studies are needed to guide optimal management strategies.

## 1. Introduction

Paraganglioma is a uncommon intra- or extra-adrenal neuroendocrine neoplasm that generates and stores adrenaline and noradrenaline. Intra-adrenal paragangliomas were previously classified as pheochromocytomas. These tumors can originate from sympathetic vs. parasympathetic autonomic ganglia, and the sympathetic one is notoriously associated with catecholamine induced (epinephrine and norepinephrine) cardiovascular complications. Patients typically exhibit symptoms associated with catecholamine excess, including palpitations, paroxysmal or sustained hypertension, headaches, anxiety, and fatigue. The diversity of these manifestations is highly variable and contingent on the genetic makeup of the tumor itself [1,2,3].

The catecholamine-induced hypertensive crisis (CHC) is a rare, life-threatening condition inducing hemodynamic instability and multiple organ failures resulting from the excessive discharge of catecholamines [4]. The management of the catecholamine-induced hypertensive crisis involves initial medical therapy, followed by appropriate alpha-blockade and fluid replenishment before surgical intervention [5]. Nevertheless, progressive multi-organ deterioration, including Takotsubo-like cardiomyopathy, cardiogenic shock, and intestinal ischemia, still occurs in some individuals despite the administration of alpha- and beta-blockers. Adrenalectomy can lead to rapid hemodynamic stabilization and recovery, even though some cases require venoarterial-extracorporeal membrane oxygenation (VA-ECMO)-assisted emergency adrenalectomy [6]. Therefore, the optimal timing of the operation during CHC remains a subject of debate [7]. However, perilous complications, such as Takotsubo-like cardiomyopathy and cardiogenic shock, have been documented and are often lethal. In this scenario, VA-ECMO might be the sole therapeutic recourse, but only a handful of instances of ECMO-managed paraganglioma-induced cardiogenic shock have been reported [8,9,10].

We present a rare and catastrophic instance of intra-adrenal paraganglioma CHC with multi-organ involvement, addressed through a multidisciplinary strategy. Additionally, we conducted a narrative literary review of published cases of paraganglioma-induced cardiogenic shock necessitating mechanical circulatory support.

## 2. Material and Methods

### 2.1. Case Presentation

A 43-year-old female presented at the emergency room of a spoke hospital with diarrhea, vague abdominal pain and few episodes of vomit. The patient did not have fever, angina or dyspnea but was referring to back pain. The previous medical history included neurofibromatosis type 1, previous cholecystectomy, right cerebral hematoma with dural artero-venous fistula treated with endovascular embolization and relapse of artero-venous fistula treated with cortical drainage.

**Figure 1:**
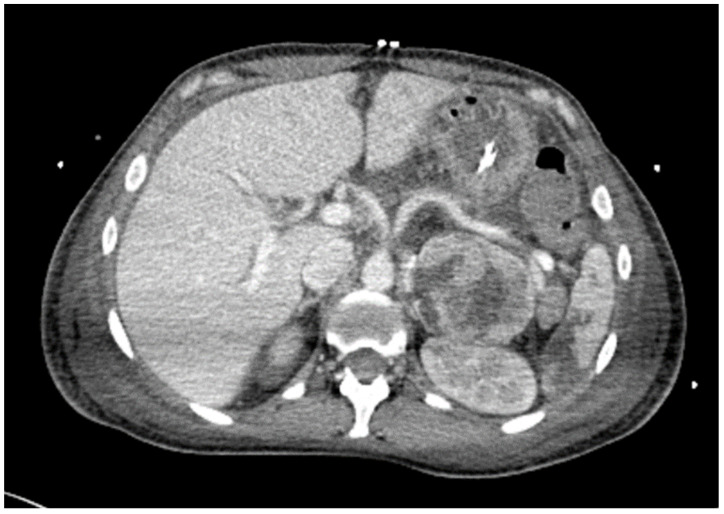
The Emergency CT scan showed a giant left adrenal tumor.

At the emergency room an abdominal ultrasonography was carried out which did not reveal any anomalies. Arterial blood gas analysis showed pH 7.2, pCO_2_ 25 mmHg, pO_2_ 79 mmHg, HCO_3_ 12 mmol/L, a marked elevated serum lactate 9.8 mg/dL compatible with metabolic acidosis. Laboratory analysis showed creatinine 1.9 mg/dL (eGFR 32 mL/min/1.73 m^2)^, White Blood Cells (WBC) 28.62 × 10^3^/mmc, Hb 15.5 g/dL, platelets 51 × 10^3^/mmc, procalcitonin 1.13 ng/mL, Troponin I (TnI) 8363 pg/mL and hepatic function in range. Tests for SARS-CoV-2 and influenza were negative but a persistent hypertensive state with systolic blood pressure peaks at 200 mmHg was present. During the clinical evaluation, the clinical condition worsened. A severe respiratory failure occurred requiring orotracheal intubation and severe hemodynamic instability requiring double vasoactive drugs support. The electrocardiogram showed supraventricular tachyarrhythmia with an average heart rate of 140 bpm, treated with adenosine with initial interruption and subsequent resumption at a high heart rate. While the echocardiogram showed a cardiac ejection fraction of 35% with akinesia of the apical segments and hyperkinesia of the basal segments. An abdomen and thoracic computerized tomography (CT) scan was performed and showed multiple pulmonary thickenings in a likely setting of heart failure associated with pneumonic superinfection with bronchopulmonary radiological image; in the abdomen, the presence of a giant left adrenal tumor of 6.4 × 5.3 cm was suspected for intra-adrenal paraganglioma. (Hounsfield Unit 127) (Figure 1) For suspected cardiogenic shock and the finding of adrenal lesion, the patient was transferred to our tertiary hospital where she was transferred to coronary intensive care unit and started on systemic support with ExtraCorporeal Membrane Oxygenation (ECMO). Urinary metanephrine and normetanephrine assays were requested; however, due to therapy with VA-ECMO, ultrafiltration, amines and the acute situation of the patient were not reliable; therefore the treatment with alpha blockers was initiated. VA-ECMO therapy lasted 6 days until discontinuation due to marked improvement in haemodynamic parameters.

**Figure 2:**
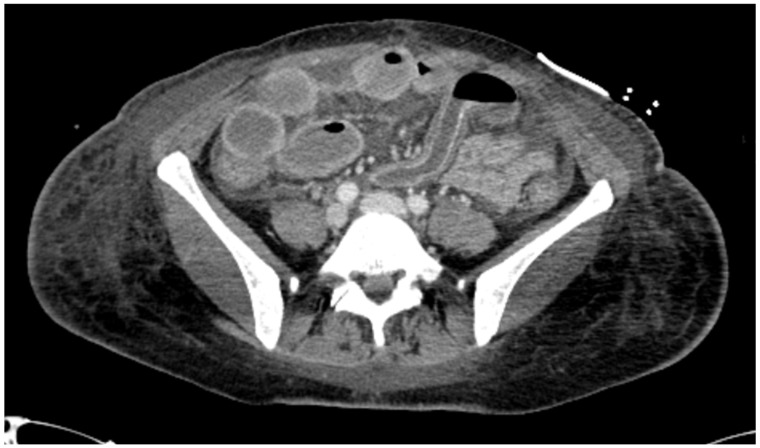
The CT scan showed diffuse bowel ischemia.

Four days after stopping ECMO therapy, the patient experienced severe abdominal pain accompanied by new hemodynamic worsening and increased lactates. Due to the persistent instability of the general clinical conditions an abdominal CT scan was performed and which raised strong doubts of intestinal ischemia. (Figure 2) An explorative laparoscopy was immediately carried out and confirmed the bowel ischemia, therefore an ileal resection, sigmoidectomy and open abdomen with application of negative pressure device were performed.

**Figure 3:**
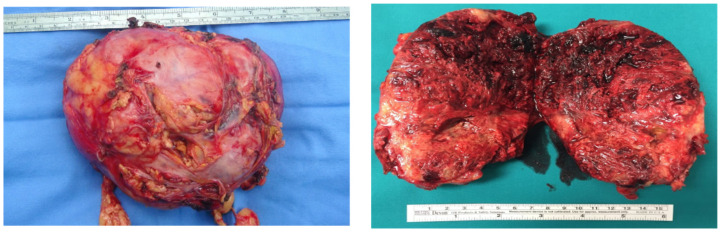
The large left intra-adrenal paraganglioma removed.

After the operation, the case was discussed at multidisciplinary setting (surgeons, anesthesiologists, radiologists and endocrinologist), and it was decided that the radiological characteristics of the adrenal gland lesion were suggestive of an intra-adrenal paraganglioma. Therefore, left adrenalectomy was indicated. After achieving hemodynamic stabilization sufficient for surgery, the patient underwent an open left adrenalectomy, followed by continued open abdominal management (Figure 3). After 72 h, further surgical revision was performed with the creation of an ileo–ileal and colon–rectal anastomosis and closure of the abdomen. After seven days, the patient’s general condition worsened again. A second surgical intervention was performed due to the dehiscence of the ileal anastomosis and perforation of the appendix. Both the anastomosis and appendix were resected, and open abdomen management with the application of a negative pressure device was initiated and completed after 48 h with an ileostomy. After about 4 days, the patient developed an acute ischemia of the left lower limb on an embolic basis and therefore intravenous sodium heparin was started. The initiation of anticoagulant therapy after about 3 days caused a massive hemoperitoneum with hemorrhagic shock induced by bleeding from collateral branch of superior mesenteric artery and retroperitoneum so a damage control surgery with packing was carried out. At new surgical abdominal look packing was removed. Despite medical therapy with noradrenaline and the surgical interventions, the state of septic shock induced by *A. Baumannii* infection worsened. After all, the patient developed a persistent Disseminated Intravascular Coagulation (DIC) even though repeated platelet, coagulation factors and fresh plasma transfusion. As a result of persistent DIC, the left lower limb underwent necrosis, and it was amputated.

Consequently, the clinical conditions deteriorated progressively and because of severe multiorgan failure the patient died after 32 days from admission to the ICU.

### 2.2. Review Methods

This narrative review aimed to summarize and describe reported cases of patients with intraadrenal paraganglioma who required mechanical circulatory support due to severe hemodynamic instability or cardiogenic shock. This comprehensive review was performed according to the methodological criteria of literature revision [11].

### 2.3. Search Strategies

We conducted a comprehensive literature search using the MEDLINE database through PubMed, as well as Google Scholar, Embase, and Scopus, covering the period from January 2013 to January 2025. The term pheochromocytoma was used because it is still the most commonly used term to indicate intra-adrenal paraganglioma. The following search terms were used: ((pheochromocytoma) AND (cardiogenic shock)), ((pheochromocytoma) AND (ECMO)), ((pheochromocytoma) AND (mechanical circulatory support)), ((cardiogenic shock) OR (adrenergic cardiomyopathy) AND (intestinal ischemia)). Additionally, we manually screened the reference lists of the included articles to identify further relevant studies.

Only English-language publications reporting individual or grouped clinical cases with available demographic and clinical data were included. Abstracts, reviews without case descriptions, and preclinical studies were excluded.

We included case reports, case series, and observational studies describing adult patients who required mechanical circulatory support (e.g., ECMO, Impella, IABP) in the setting of paraganglioma-induced cardiogenic shock. Studies involving pediatric or neonatal patients, as well as those lacking outcome data, were excluded.

From each eligible study, we extracted the following data when available: year of publication, number of patients, age, sex, tumor location (right adrenal, left adrenal, bilateral adrenal glands, or extra-adrenal/paraganglioma), initial left ventricular ejection fraction (LVEF), type and duration of mechanical circulatory support, interval between ICU admission and surgery (if performed), and in-hospital mortality. Cases involving pregnancy were also noted. Echocardiographic findings and other relevant clinical data were collected when reported.

Given the descriptive and heterogeneous nature of the included studies, which consisted primarily of individual case reports and small case series, no formal risk of bias assessment was conducted because since they are all case reports or case series, the methodological applicability is lost. These types of studies inherently carry a high risk of publication and selection bias, and their findings should be interpreted accordingly.

The literature search was performed independently by two authors (AG and MM). Any discrepancies between the reviewers were discussed and solved by consensus.

## 3. Results

The literature search identified a total of 42 eligible articles published between January 2013 and January 2025, reporting on 68 patients who developed cardiogenic shock secondary to paraganglioma and one right adrenal Ewing’s Sarcoma and required mechanical circulatory support. Of these, 36 were single-patient case reports, and 6 were small case series, with a maximum of 14 patients. Data are summarized in Table 1 [4,6,12,13,14,15,16,17,18,19,20,21,22,23,24,25,26,27,28,29,30,31,32,33,34,35,36,37,38,39,40,41,42,43,44,45,46,47,48,49,50].

The mean age of patients was 39.8 ± 12.5 years. Among the 69 patients with available sex data, 46 (66.7%) were female—including 4 pregnant patients—and 23 (33.3%) were male. Tumor location was reported as follows: 21 patients had right adrenal paraganglioma, 25 had left adrenal paraganglioma, 2 had bilateral adrenal paraganglioma, and 6 had extra-adrenal paragangliomas. One patient was diagnosed with a primary adrenal Ewing sarcoma. In 14 cases, the side of the intra-adrenal paraganglioma was not reported.

Initial LVEF was reported in 24 studies. The mean initial LVEF was 12.5%, with values ranging from 5% to 25%, consistent with severe catecholamine-induced cardiomyopathy.

Mechanical circulatory support was most commonly provided via ECMO, either alone or in combination with other devices. Among the 34 cases in which duration was reported, the median duration of support was 6 days, ranging from 1 to 16 days. ECMO alone was used in the majority of cases, while a minority required combined support with IABP or Impella.

One patient was supported with a TandemHeart device, and four patients underwent cardiopulmonary bypass to facilitate surgical resection of the paraganglioma.

Surgical removal of the tumor was performed after weaning from mechanical circulatory support in 16 patients (20.2%), while 58 patients (73.4%) underwent surgery while still on support. Two patients (2.5%) died before surgery, and one patient (1.3%) refused surgical treatment. In the remaining cases, the timing of surgery was not reported.

The interval from ICU admission to surgery was available in 23 studies, with a mean of 28 days (range: 10–66 days). In two of these cases, surgery was not performed—one due to clinical deterioration and one due to patient refusal.

Overall, 12 patients died out of 69 cases with available outcome data, corresponding to a hospital mortality rate of 17.4%.

## 4. Discussion

The catecholamine-induced hypertensive crisis, while a rare ailment, possesses the potential to be fatal owing to its cardiovascular complications, with an occurrence rate until 70% [1,51]. The clinical manifestation of CHC is broadly diverse and non-specific, and the quintessential triad—comprising episodic headaches, diaphoresis, and tachycardia—is observed in only a minority of patients [8]. Whitelaw et al. [9] categorized CHC into two distinct types. Type A is characterized as a less severe crisis without sustained hypotension, whereas Type B is described as a critical presentation involving persistent hypotension, shock, and multiorgan dysfunction, as evidenced in our case. Several hypotheses have been put forth to elucidate the pathophysiology of cardiovascular failure. These include coronary vasospasm, triggered by alpha-1 stimulation (also known as Takotsubo cardiomyopathy) [10], elevated myocardial oxygen demand due to tachycardia and increased afterload, and the direct myocytotoxic impact of heightened catecholamine levels. The profuse and overwhelming release of catecholamines is the underlying basis, through a similar pathological pathway, for causes of non-occlusive intestinal ischemia, as seen in our patient.

Addressing a catecholamine-induced hypertensive crisis can be formidable. CHC represents a critical and lethal emergency, necessitating organ-specific mechanical support, which encompasses mechanical ventilation (85%), circulatory support (vasoactive drugs in 68% and ECMO in 41%), and renal replacement therapy (24%) [2]. Despite the use of the rescue methods noted above, the mortality rate for CHC remains substantial (15–30%) [9].

Alpha antagonists and beta-blockers constitute the primary pharmacological intervention, yet their administration in patients experiencing hemodynamic instability can prompt certain apprehensions. Furthermore, elevated endogenous levels of catecholamines may induce sympathetic receptor desensitization. Vasopressors and inotropes commonly employed to manage cardiogenic shock (including adrenaline, noradrenaline, and dobutamine) might be less efficacious, or could even aggravate paraganglioma-induced cardiac dysfunction [12]. Myocardial stunning, resulting from catecholamine surplus, is largely reversible if promptly and appropriately managed. In instances unresponsive to medical therapy, mechanical circulatory support devices have demonstrated efficacy in stabilizing hemodynamics and facilitating recuperation. In addition to alpha and beta adrenreceptor blockade, other agents can be considered, such the metyrosine (inhibitor of tyrosine hydroxylate, the rate limiting enzyme in catecholamine biosynthesis),the ivabradine for catecholamine induced tachycardia and the recent data on belzutifan use in catecholamine induced hypertension and its potential use in emergent situation given the rapid response as follow up data from the study label showed up to 32% of patients treated with belzutifan had reduction and de-escalation in their hypertension treatment [52].

However, the utilization of ECMO in paraganglioma-induced cardiogenic shock continues to be a subject of ongoing discussion. Recent investigations [49,53] have documented the successful deployment of ECMO to rescue patients with severe cardiopulmonary failure, achieving survival rates of 87%, but these data are confined to case series and observational reports.

Our patient presented signs of adrenergic crisis and rapidly progressed to multiorgan failure. The initial echocardiographic pattern—apical akinesia and basal hyperkinesia—was compatible with Takotsubo-like cardiomyopathy. A left adrenal mass consistent with intra-adrenal paraganglioma was detected, but metanephrine levels were unreliable due to the critical state and ongoing ECMO therapy. Based on clinical and radiological findings, alpha-blockade was empirically initiated. Despite ECMO support and surgical adrenalectomy, the clinical course was complicated by bowel ischemia, sepsis, disseminated intravascular coagulation, limb ischemia, and ultimately death due to multiorgan failure.

Our case illustrates several critical challenges in managing CHC: the difficulty of biochemical confirmation during acute shock, the potential for ECMO to stabilize patients while awaiting tumor resection, and the high risk of systemic complications. While our patient did not survive, a literature analysis of 69 similar cases revealed a hospital mortality rate of 17.4%, with most survivors undergoing adrenalectomy during or shortly after ECMO support. These findings emphasize that early recognition, prompt hemodynamic support, and surgical tumor removal are essential for improving outcomes, but also that complex complications—as intestinal ischemia and septic shock—remain major threats.

However, the optimal timing of adrenalectomy remains a matter of debate. Current literature often lacks clarity on the interval between ICU admission and surgery, and few reports specify whether surgery occurred during or after ECMO weaning. While elective paraganglioma surgery typically requires preoperative adrenergic blockade to prevent perioperative cardiovascular complications [54,55,56], such preparation is often not feasible in emergency settings. Performing surgery during active ECMO support may offer a safety net against perioperative cardiovascular collapse from catecholamine surges, but it also introduces risks, including the need to suspend anticoagulation—which increases the chance of thrombosis and ECMO circuit complications. Additionally, proceeding to surgery without proper alpha-blockade may expose patients to hypertensive crises, cerebrovascular complications, or severe vasoplegic shock due to sudden catecholamine withdrawal.

Our case was further complicated by intestinal ischemia requiring emergent laparotomy and bowel resection. Given the open abdomen and temporary hemodynamic stabilization, we opted to proceed with adrenalectomy as part of a staged surgical strategy. While this approach cannot be generalized, it highlights the need for flexible, individualized decision-making in complex cases. To our knowledge, no other published case describes CHC complicated by intestinal ischemia requiring laparotomy. In this context, we prioritized removal of the suspected source of sepsis through damage control surgery, followed by delayed definitive procedures, including adrenalectomy and abdominal wall closure. This multidisciplinary approach aligned with current strategies for critically ill surgical patients [57], though further research is needed to validate timing and sequence of interventions in PMC.

Given the rarity of this condition, the role of high-volume centers with expertise in adrenal tumors, anesthesiology, intensive care, and cardiology should be emphasized. Timely referral and centralized care may improve outcomes, particularly when MCS and advanced surgical strategies are needed. Nevertheless, available evidence on ECMO in this context is limited to case reports and small series, reinforcing the urgent need for higher-quality studies and multicenter registries.

The main strength of this work lies in the comprehensive analysis of a rare and highly complex case, supported by a narrative synthesis of 69 published cases of paraganglioma induced cardiogenic shock requiring mechanical circulatory support. To our knowledge, this is the first report to describe intestinal ischemia as a presenting complication in this setting, contributing to the clinical understanding of CHC.

However, several limitations must be acknowledged. First, the literature review was based exclusively on case reports and case series, with inherent risks of publication bias and incomplete reporting. The descriptive nature of the data limited our ability to perform any statistical or comparative analysis. Second, our conclusions are based on a single case, and caution must be taken in extrapolating management strategies to broader populations. Finally, the lack of standardized reporting on timing of surgery and outcomes in existing studies limits the generalizability of proposed approaches.

## 5. Conclusions

The catecholamine-induced hypertensive crisis remains a diagnostic and therapeutic challenge, particularly when complicated by cardiogenic shock and multiorgan dysfunction. Mechanical circulatory support, especially ECMO, can provide a vital bridge to stabilization and definitive surgical treatment, although the optimal timing of tumor excision remains unclear. This case highlights the potential severity of catecholamine excess and the complexity of managing such patients, especially when complications like intestinal ischemia occur. While our findings are limited by the descriptive nature of a single case and a literature review based on case reports, they underscore the importance of early recognition, multidisciplinary management, and centralization of care. Further studies with higher levels of evidence are needed to guide timing, perioperative strategies, and the role of ECMO in this rare but life-threatening condition.

## Figures and Tables

**Table 1:** Results of current comprehensive literature review.

First Author	Year	Patients, *n*	Mean Age (Years)	Sex	Adrenal Lesion	Initial LVEF (%)	Mechanical Circulatory Support; Duration (Days)	ICU-Admission -to-Surgery Interval (Days)	Hospital Mortality, n (%)
Kaese [13]	2013	1	43	M	Right adrenal PHEO	NR	ECMO; 9	NR, after ECMO weaning	0 (0%)
Law [14]	2013	1	23	F	Right adrenal PHEO	5	ECMO; 1	NR, after ECMO weaning	0 (0%)
Shawa [15]	2014	1	38	F	Left adrenal PHEO	15	Tandem Heart; 1	26	0 (0%)
Riester [4]	2015	2	18	F	Right adrenal PHEO	NR	Impella; 2	No surgery	2 (100%)
Chao [16]	2015	4	25	1 F, 3 M	3 Left adrenal PHEO, 1 Right adrenal PHEO	NR	ECMO, NR	NR, after ECMO Weaning (1 refused surgery)	4 (100%)
Zhou [17]	2015	1	35	M	PARA	20	ECMO and IABP; 16	NR, after ECMO Weaning	0 (0%)
Flam [18]	2015	1	46	F	Left adrenal PHEO	15	ECMO; 15	66	0 (0%)
Vagner [19]	2015	1	55	F	Left adrenal PHEO	15	Impella then ECLS; 2	56	0 (0%)
Kodama [20]	2016	1	37	F	Right adrenal PHEO	10	ECMO; NR	NR, before ECMO weaning	0 (0%)
Dang Van [21]	2016	1	57	M	PARA	10	ECMO;7	NR, concomitant from the ECMO implantation	0 (0%)
Hekimian [22]	2016	9	43	7 F, 2 M	4 Right adrenal PHEO, 5 Left adrenal PHEO	16 (median)	ECMO; 15 (median)	35 (median)	3 (33.3%)
van Zwet [23]	2016	1	27	F (Pregnant)	Left adrenal PHEO	NR	ECMO; 7	22	0 (0%)
Mita [24]	2016	1	29	F, (Pregnant)	Left adrenal PHEO	Nr	IABP, 4; ECMO, 6	60	0 (0%)
Bouabdallaoui [25]	2017	1	40	F	Left adrenal PHEO	15	ECMO; 5	28	0 (0%)
Sauneuf [12]	2017	14	43	7 F, 7 M	Nr	Nr	ECMO; 4 (median)	10 (median)	3 (21.4%)
Kang [26]	2019	1	31	M	Right adrenal PHEO	10	ECMO; 3	NR, after ECMO Weaning	0 (0%)
Mierke [27]	2019	1	47	M	Left adrenal PHEO	NR	Impella + ECMO; 3	33	0 (0%)
Huang [28]	2019	1	58	F	PARA	NR	ECMO; 5	NR, after ECMO Weaning	0 (0%)
Garla [29]	2019	1	55	F	Right adrenal PHEO	11	IABP + ECMO; 5	21	0 (0%)
Min [30]	2019	1	31	F	Right adrenal PHEO	6	ECMO; NR	28	0 (0%)
Kiamanesh [31]	2019	1	45	F	Right adrenal PHEO	NR	ECMO; 3	NR, after ECMO Weaning	0 (0%)
Montalto [32]	2020	1	28	F	Left adrenal PHEO	7	ECMO, 4 + Impella, 6	21	0 (0%)
Dominedò [33]	2020	1	26	F	Bilateral Adrenal Pheo (MEN 1)	NR	ECMO; 14	35	0 (0%)
Dominedò [34]	2020	1	28	F	Left adrenal PHEO	11	ECMO, 4 + Impella, 5	18	0 (0%)
Attisani [35]	2021	2	43	1 F, 1 M	1 Left adrenal PHEO, 1 Right adrenal PHEO	NR	ECMO; 6	13 (median)	0 (0%)
Yang [36]	2021	1	40	M	Bilateral Adrenal Pheo	NR	ECMO; 8	NR, after ECMO Weaning	0 (0%)
Nakayama [37]	2021	1	59	F	Left adrenal PHEO	12	IABP, 4 + ECMO, 3	39	0 (0%)
Chen [38]	2021	1	55	M	PARA	8	ECMO; 6	23	0 (0%)
Levin [39]	2021	1	33	F	PARA	NR	ECMO; 4	42	0 (0%)
Lüsebrink [40]	2021	1	19	F	PARA	15	ECMO; NR	NR, after ECMO Weaning	0 (0%)
Myatt [41]	2021	1	36	F (pregnant)	Left adrenal PHEO	13	ECMO; NR	NR, after ECMO Weaning	0 (0%)
Choudhary [6]	2021	1	30	M	Right adrenal PHEO	9	ECMO; 4	37	0 (0%)
Wang [42]	2022	1	20	F	Right adrenal Ewing’s Sarcoma	NR	ECMO; 11	NR	0 (0%)
Lyu [43]	2022	1	54	F	Right adrenal PHEO	20	ECMO; 6	NR, after ECMO Weaning	0 (0%)
Luo [44]	2022	1	50	F	Left adrenal PHEO	NR	ECMO; 6	28	0 (0%)
Nakayama [37]	2023	1	59	F	Left adrenal PHEO	NR	ECMO; NR	30	0 (0%)
Fennell [45]	2023	2	42	2 F	2 Right adrenal PHEO (1 case NF1)	15 (median)	ECMO; 6	41	0(%)
Xie [46]	2023	1	46	F	Left adrenal PHEO	NR	ECMO; 7	11	0 (0%)
Zhong [47]	2023	1	32	M	Right adrenal PHEO	8	ECMO; NR	NR, after ECMO Weaning	0 (0%)
Tran [48]	2023	1	54	M	Right adrenal PHEO	25	ECMO; 7	NR, after ECMO Weaning	0 (0%)
Park [49]	2024	1	29	F (pregnant)	Left adrenal PHEO	NR	ECMO; 8	NR, after ECMO Weaning	0 (0%)
Chen [50]	2024	1	64	F	Left adrenal PHEO	20	ECMO; NR	NR, after ECMO Weaning	0 (0%)

## Data Availability

The data reported are available in our databases and accessible upon request to the corresponding author.
